# Teaching communication skills in clinical settings: comparing two applications of a comprehensive program with standardized and real patients

**DOI:** 10.1186/1472-6920-14-92

**Published:** 2014-05-09

**Authors:** Irene P Carvalho, Vanessa G Pais, Filipa R Silva, Raquel Martins, Margarida Figueiredo-Braga, Raquel Pedrosa, Susana S Almeida, Luís Correia, Raquel Ribeiro-Silva, Ivone Castro-Vale, Ana Teles, Rui Mota-Cardoso

**Affiliations:** 1 Medical Psychology Unit, Department of Clinical Neurosciences and Mental Health, School of Medicine, University of Porto, Porto, Portugal; 2 Psicologia Médica, Faculdade de Medicina, Universidade do Porto, Al. Prof. Hernâni Monteiro, 4200-319 Porto, Portugal

**Keywords:** Communication program, Competence, Healthcare professionals, Standardized patients, Real patients, Replication

## Abstract

**Background:**

Communication is important for the quality of clinical practice, and programs have been implemented to improve healthcare providers’ communication skills. However, the consistency of programs teaching communication skills has received little attention, and debate exists about the application of acquired skills to real patients. This study inspects whether (1) results from a communication program are replicated with different samples, and (2) results with standardized patients apply to interviews with real patients.

**Methods:**

A structured, nine-month communication program was applied in two consecutive years to two different samples of healthcare professionals (25 in the first year, 20 in the second year). Results were assessed at four different points in time, each year, regarding participants’ confidence levels (self-rated), basic communication skills in interviews with standardized patients, and basic communication skills in interviews with real patients. Data were analyzed using GLM Repeated-Measures procedures.

**Results:**

Improvements were statistically significant in both years in all measures except in simulated patients’ assessment of the 2008 group. Differences between the two samples were non-significant. Differences between interviews with standardized and with real patients were also non-significant.

**Conclusions:**

The program’s positive outcomes were replicated in different samples, and acquired skills were successfully applied to real-patient interviews. This reinforces this type of program structure as a valuable training tool, with results translating into real situations. It also adds to the reliability of the assessment instruments employed, though these may need adaptation in the case of real patients.

## Background

Studies consistently report improvements in communication skills among participants in programs teaching these competencies in healthcare settings [1-3]. The efficacy of the programs may vary depending on the teaching methods employed. Programs with modules divided into three sections are considered the exemplary practice for teaching these skills. They comprehend cognitive, modeling, and behavioral components, corresponding respectively to didactic, illustrational (e.g., through videos and other examples) and small group role-playing with feedback [4,5]. Course structure has been offered as an explanation also for the long-term effects observed in communication skills acquired after a program, where the advantages of problem-based over lecture-based curricula are highlighted [6,7]. In addition, courses with more hours of training that are intensive, practice-oriented and skills-focused tend to result in higher levels of change [1,3,5]. Less attention has been paid to the consistency of particular programs in effecting changes with different applications and different samples, that is, program replicability – a point also made in previous research highlighting this limitation in the various reviewed studies [3].

Improvements in communication skills after programs aimed at these competences are noticeable in participants’ subjective sense of confidence in their clinical practice [8,9] and reflected in externally rated encounters with patients [10-12]. Programs commonly use standardized or simulated patients (SPs) to train and evaluate communication skills [13-15]. Though these two terms are frequently treated interchangeably, standardized patients are simulated patients trained to deliver identical, standardized performances [16-19], and both have been shown to provide reliable ratings of students’ competence [1,15,20]. Most importantly, however, is the application of acquired skills to real-life situations, with real patients (RPs).

Studies have shown that improvements in communication skills after a program are observed in encounters with both simulated and real patients [11,21,22], but the more important changes happened in SPs’ interviews. Professionals’ levels of performance with RPs are lower than with SPs [23]. SPs have many recognized advantages over RPs (including their potential for a more objective assessment of interpersonal communication [19,20] and their knowledge of the situation, which leads to more focused feedback regarding students’ performances [24]). However, SPs recreate artificial situations that may lack the realism inherent to encounters with RPs [19,25,26]. The skills healthcare professionals employ in one and the other situation may thus be different [26] and, even if adequate, they may escape checklists used to evaluate performance [25]. Though research has reported that students react in similar ways to simulated and real patients [27], interacting with SPs may thus be different than interacting with RPs in real situations in terms of applying acquired communication skills. Instruments that adapt well to one situation may not fully adapt to the other.

Since effective communication has been shown to be relevant to clinical practice [28-30], and programs are created to improve communication between healthcare professionals and their patients, it is important to assess the consistency of these programs in operating changes and the extent to which these changes transfer to real situations with RPs, for whom they are intended. To these ends, the current study has two goals: (1) to inspect whether the results obtained with a structured, comprehensive program teaching communication skills can be replicated with a different sample of professional practitioners (to examine the effects of aspects such as chance, or sample characteristics, on the results), and (2) to examine whether results obtained with SPs in controlled situations apply to interviews with RPs in real situations.

## Methods

### The program

The program had a duration of nine months and was offered in 2008 and, again, in 2009 as a post-graduate program on communication skills. Each year, the same content was taught by the same faculty members, following the same structure and sequence. The program has been described in detail in a previous work [31] and is summarized in Table 1. The current study targets basic communication skills, the focus of the initial three months of the program.

**Table 1:** Communication skills program: sequence and evaluation procedures throughout nine months

**The program**			
**Months 1-3**	**Months 4-6**		Months 7-9*
**Basic communication skills**	**Advanced communication skills**		**Self-awareness, Self-help, Communication in special situations**
- Structuring an interview	- Dealing with strong emotions		- Patients unable to speak
- Patient-centered interview	- Breaking bad news		- Children, adolescents and the elderly
- Doctor-centered interview	- Motivational interviewing		- Families
- Non-verbal behavior			- Patients suffering from depression and anxiety
- Building a clinical relationship			
**Evaluation procedures**			
**T0**	**T1**	**T2**	**T3**
**Before the program**	**Month three**	**Month six**	**End of the program**
**Basic communication skills**			
Interview with SP	Interview with SP	Interview with SP	Interview with SP
- SEGUE rated by 2 faculty members	- SEGUE rated by 2 faculty members	- SEGUE rated by 2 faculty members	- SEGUE rated by 2 faculty members
- ICSC rated by actor (SP)	- ICSC rated by actor (SP)	- ICSC rated by actor (SP)	- ICSC rated by actor (SP)
	Interview with RP	Interview with RP	Interview with RP
	- SEGUE rated by 1 faculty member	- SEGUE rated by 1 faculty member	- SEGUE rated by 1 faculty member
**Participants’ confidence in their communication skills**			
- Self-confidence		- Self-confidence	- Self-confidence

*The program’s final three-month period has a lesser emphasis on practice.

SP: standardized patient. RP: real patient.

Classes met twice a week for five hours each day and typically included theoretical presentations of the materials, role-modeling through video viewing and discussion, and role-playing in small groups of peers (six or seven students), with feedback by both peers and faculty. Participants were divided into the smaller groups according to their professional backgrounds, to maximize group heterogeneity and exposure to professional diversity. Two faculty members per group tutored the same students throughout the program, encouraging participation and discussion. The role-playing practice represented a substantial part of the program (about 67 percent of the course load) and used previously written scripts based on cases of patients from the faculty’s and the students’ clinical experiences.

### Procedures

In this observational study, participants were evaluated at four moments in time: before the program (T0); three months into the program, at the end of the basic communication skills section (T1); six months into the program, at the end of the advanced skills section (T2); and three months later, at the end of the program (T3). In each moment, participants conducted a 25-minute first interview with SPs. All interviews were videotaped with participants’ consent. Participants were also asked to record a 25-minute first interview with one of their RPs, in a real situation, at T1, T2 and T3.

Participants were evaluated on their communication performances in the videotaped interviews with SPs (by two trained faculty members per interview and by the SP after the interview) and with RPs (by one trained faculty member per interview). They were additionally evaluated on their (self-rated) levels of confidence in conducting clinical encounters at T0, T2 and T3. Participants were informed that, with the exception of the interview at T0, all interviews with SPs and with RPs counted towards their grades. Participation was voluntary, and data were used for research purposes with participants’ consent. The study was approved by the Ethics Committee for Health of S. João Hospital/School of Medicine of Porto, and complies with the Declaration of Helsinki.

### Standardized patients

The interviews in 2008 and 2009 used the same five professional actors (three males and two females) from one theatre company who met with the students during these evaluation times exclusively. The actors shared the same ethnic background as the students (all Caucasian). Three were in their thirties, one male was in his late twenties and one male was in his fifties. Each actor met with an average of three to eight participants per evaluation time each year.

The actors had no previous experience as SPs, though two had majored in psychology and a third had attended several workshops dealing with communication in medical settings. The team of faculty members (all trained and experienced teachers in communication skills) coached the actors on the purposes of the interviews, on the scripts of ‘patients’ created for the SPs and on the specific aspects of the encounters, such as the amount and kind of “talking” and “acting” they should do for the purposes of these interviews. Each script was reviewed with each actor, and possible responses and interactions rehearsed. The initial preparation took about two hours with each actor, plus an average of over two hours actors reported spending with each script at home. At each evaluation point, actors were again briefed on the scripts, the acting and the purposes of the program. Further discussions of the cases and the interviews occurred throughout the programs on demand. Additionally, faculty members systematically monitored and gave SPs feedback about their performances throughout the programs in order to maintain acting quality and consistency, and keep it within the evaluation goals.

Written by faculty members with clinical experience, the scripts featured situations based on real cases of patients. Tailored to the actors (each actor had his or her own scripts) and adapted to the professional areas of the participants in this study, all scripts followed the same format and contained detailed information on patients’ personal circumstances (e.g., age, occupation), sets of symptoms (and associated emotions, beliefs and impact in daily life), health history, family health history, life style (e.g., physical activity, alcohol consumption), and psychosocial aspects (e.g., life satisfaction, preoccupations, support net). Since the main goal of the study was communication, actual clinical symptoms were consistently kept simple across scripts, namely to ensure equivalent situations across the different professional areas involved.

### Instruments

Faculty members assessed participants’ communication skills in interviews with SPs and with RPs using the SEGUE framework [32]. SPs rated students’ performances after each interview using the Interpersonal and Communication Skills Checklist (ICSC) [33]. Participants rated their own levels of confidence in conducting clinical encounters using a modified version of Smith et al.’s self-efficacy questionnaire [8]. All instruments were translated into Portuguese, and item clarity was individually checked with respondents in the initial interviews.

These instruments, as well as the rationale for their choice, are described elsewhere [8,15,31-34]. In summary, the SEGUE framework is a 25-item (yes/no) checklist designed by Makoul to facilitate teaching and assessment of critical communication tasks [32]. Easy to use, it has also demonstrated acceptable psychometric characteristics in varied contexts over the years [32,34].

The ICSC was developed by the eight medical schools in the New York City Consortium for Clinical Competence. It is a 17-item (yes/no) checklist designed to assess interpersonal and communication skills that has shown acceptable psychometric properties [33].

Developed to assess residents’ attitudes towards psychosocial skills used in medical care, Smith et al.’s questionnaire is a 38-item, 7-point Likert scale that evaluates self-efficacy [8]. The original instrument contains items that assess domains unrelated to our curriculum, and we used the 17 items that do correspond to our learning contents, adapting them to our program. Students evaluated their sense of self-efficacy regarding clinical communication skills (e.g., How confident are you that you can refrain from interrupting the patient? How confident are you that you can identify unexpressed feelings?) in a 7-point Likert scale (from 1 = not at all confident to 7 = totally confident). For the purposes of this study, only the 10 initial items, focusing on basic communication skills, were used (the remaining seven are directed at advanced skills and special situations and were excluded).

### Participants

Twenty-five healthcare professionals participated in the program in 2008 and another 20 enrolled in 2009. The two groups are comparable in several ways: professional background composition (physicians, nurses, clinical psychologists, physiotherapists, speech therapists, etc., present in both groups in similar proportions, the first two above-mentioned professions making up about 68% of the 2008 group and 65% of the 2009 group), professional experience (mean of approximately three years of practice in both groups, representing the beginning of participants’ careers), and gender (two males in 2008, two males in 2009).

### Analyses

For the SEGUE framework, and for the ICSC, the score was the percentage of items checked ‘yes’ in each interview. The mean of the scores given by the two different SEGUE raters to interviews with SPs was calculated and used. For the measure of confidence, the mean of the ratings (in the Likert scale) was computed for the used items. Total scores for each measurement time were obtained by calculating the mean of all students’ scores in each instrument.

To address the first goal of this study, GLM Repeated-Measures procedures were conducted in PASW (Predictive Analysis Software) to inspect changes over time in the measures of confidence and of performance (SEGUE and ICSC) with SPs, and to examine differences between samples, following a factorial plan of 2 × 4 – (2008/2009 group) × (T0/T1/T2/T3 evaluation times). Bonferroni correction was used for multiple comparisons (alpha = 0.01).

In order to assess whether changes with the program transfer to real situations with RPs (the second goal of this study), another GLM Repeated-Measures procedure was conducted on the measure of performance (SEGUE) according to a factorial plan of 2×3 – (SPs/RPs) × (T1/T2/T3 evaluation times). Participants’ interviews with RPs were added to their SPs’ interviews, leading to a database of 90 subjects. Because the instruction in both situations asked for a first encounter with a patient, students who conducted other types of encounters with RPs were excluded. Thus procedures were carried out for a total n = 70.

## Results

### Program replicability: the 2008 and 2009 groups

**Figure 1:**
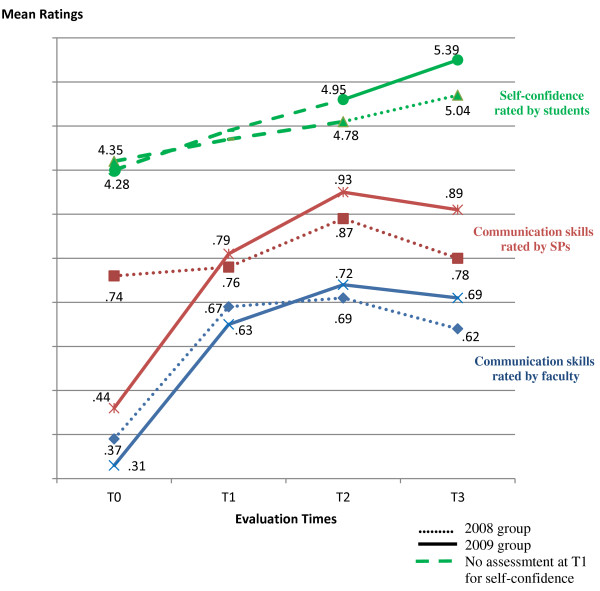
Participants’ communication skills and self-confidence throughout the program: means for the 2008 and the 2009 samples.

Performances follow similar patterns in the 2008 and 2009 groups (Figure 1). The 2009 group scored lower in all three measures before entering the program (at T0), and reached higher values throughout its course also in all three measures, than the 2008 group. Between-subjects tests show that apparent differences between the two groups throughout the program are statistically non-significant.

Effects of taking the course assessed by SPs are statistically significant for the 2009 group (as shown by within-subjects tests, including corrected Greenhouse-Geisser, Huynh-Feldt and Lower-bound procedures, *F*(3, 54) = 24.79, *p* < 0.01), but not for the 2008 group (Table 2). Within-subjects simple contrasts for SPs’ assessments of the 2009 group show significant differences between performances before the course (at T0) and each measured time after: at T1 (*F*(1, 18) = 19.14, *p* < 0.01), at T2 (*F*(1, 18) = 45.75, *p* < 0.01) and at T3 (*F*(1, 18) = 30.90, *p* < 0.01). By contrast, SPs already rated 2008 group performances at high levels before the beginning of the program, approximating them to their subsequent evaluations at T1, T2 and T3.

**Table 2:** Participants’ communication skills and self-confidence assessed throughout the program in the 2008 and in the 2009 classes (differences from T0)

**Competence (Raters)**	**Class**	**Evaluation times**			
		**T0**	**T1**	**T2**	**T3**
		**Mean**	**Mean**	**Mean**	**Mean**
		**(SD)**	**(SD)**	**(SD)**	**(SD)**
Communication skills with SPs (assessed by faculty^a^)	2008	.37	.67**	.69**	.62**
		(.15)	(.17)	(.14)	(.17)
	2009	.31	.63**	.72**	.69**
		(.13)	(.15)	(.12)	(.10)
Communication skills with SPs (assessed by SPs^b^)	2008	.74	.76	.87	.78
		(.24)	(.23)	(.16)	(.22)
	2009	.44	.79**	.93**	.89**
		(.31)	(.17)	(.08)	(.13)
Self-confidence^c^ (assessed by participants)	2008	4.37	__	4.78	5.05**
		(.74)		(.72)	(.88)
	2009	4.28	__	4.95**	5.39**
		(.95)		(.56)	(.61)

**p < 0.01.

^a^Using the SEGUE framework (‘yes/no’ checklist).

^b^Using the ICSC (‘yes/no’ checklist).

^c^Using the confidence instrument, self-rated on a 7-point Likert scale (1 = not at all confident to 7 = totally confident).

Effects of taking the course on self-confidence are statistically significant for the 2009 group (as shown by within-subjects tests, including corrected Greenhouse-Geisser, Huynh-Feldt and Lower-bound procedures, *F*(2, 36) = 17.61, *p* < 0.01), as well as the 2008 sample (shown by within-subjects tests, including corrected Greenhouse-Geisser and Huynh-Feldt procedures, *F*(2, 40) = 6.90, *p* < 0.01). At the end of the program (T3), participants were significantly more confident in their communication skills in clinical interviews than before taking it (2008 group, *F*(1, 20) = 10.17, *p* < 0.01; 2009 group, *F*(1, 18) = 24.76, *p* < 0.01), and the increase in confidence was statistically significant also at T2 for the 2009 group (*F*(1, 18) = 11.17, *p* < 0.01), though it was non-significant for the 2008 sample, reflecting the former group’s greater change with the program, mentioned above. Pairwise comparisons further indicate that, for the 2009 group, the change between T2 and T3 is also statistically significant (mean difference of 0.45, *p* = 0.009), though it is non-significant for the 2008 group, with its less pronounced increase.

Effects of the program on communication skills assessed by faculty are statistically significant for the 2009 sample (as shown by within-subjects tests, including corrected Greenhouse-Geisser, Huynh-Feldt and Lower-bound procedures, *F*(3, 57) = 48.69, *p* < 0.01), as well as the 2008 group (as shown by within-subjects tests, including corrected Greenhouse-Geisser, Huynh-Feldt and Lower-bound procedures, *F*(3, 72) = 40.31, *p* < 0.01). Comparing with T0, the increase in communication skills was statistically significant for the two samples at T1 (2008 group, *F*(1, 24) = 68.52, *p* < 0.01; 2009 group, *F*(1, 19) = 46.18, *p* < 0.01), T2 (2008 group, *F*(1, 24) = 104.83, *p* < 0.01; 2009 group, *F*(1, 19) = 117.85, *p* < 0.01) and T3 (2008 group, *F*(1, 24) = 44.02, *p* < 0.01; 2009 group, *F*(1, 19) = 93.88, *p* < 0.01) (Table 2).

Pairwise comparisons indicate that the decline observed by both external observers and SPs in communication skills at T3 is statistically non-significant, when compared with T2, for the 2008 as well as the 2009 sample (as are all other differences between T1, T2 and T3 for the two samples, whether assessed by external observers or SPs). As mentioned before, in both samples, the change in competence at T3 remains statistically significant, comparing with T0, despite the decline (except for SPs’ assessment of the 2008 group, where, as mentioned earlier, no change reached statistical significance).

### Comparison between standardized- and real-patient scores

**Figure 2:**
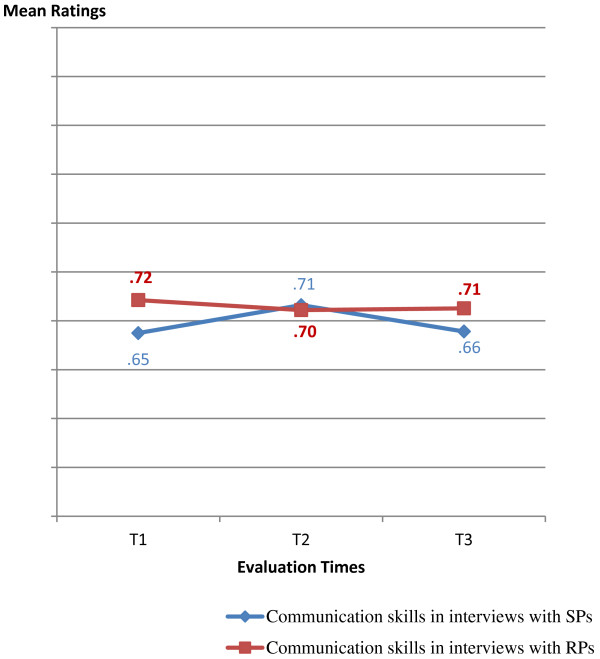
Participants’ communication skills in interviews with SPs and in interviews with RPs throughout the program (rated by faculty).

Results show the numerical similarity between ratings for SPs and for RPs throughout the program (Table 3).Participants’ communication skills reach the highest scores at T1 for RPs, with a slight (statistically non-significant) decline afterwards, whereas for SPs, the highest point occurs at T2, with a slight (statistically non-significant) decline at T3 (Figure 2). Differences between SPs’ and RPs’ interviews are statistically non-significant.

**Table 3:** Participants’ communication skills assessed throughout the program in interviews with RPs and with SPs

**Competence (Raters)**	**Patients**	**Evaluation times**		
		**T1**	**T2**	**T3**
		**Mean**	**Mean**	**Mean**
		**(SD)**	**(SD)**	**(SD)**
Communication skills (assessed by faculty^a^)	RPs	.72	.70	.71
		(.11)	(.15)	(.14)
	SPs	.65	.71	.66
		(.16)	(.12)	(.14)

^a^Using the SEGUE framework (‘yes/no’ checklist).

## Discussion

### Program replicability: the 2008 and 2009 samples

Regarding the first goal of this study, the analyses indicate that the positive outcomes of this structured, comprehensive training program are replicated in different samples in two different years. These positive outcomes are reflected, each year, in statistically significant increases in confidence, self-rated by participants, and in communication skills, assessed by external observers and by SPs, with between-group effects indicating that no differences exist overall between the 2008 and the 2009 groups throughout the program. The slight decay observed for both the 2008 and the 2009 groups at T3 has no statistical significance in either external observers’ or SPs’ assessments. These findings substantiate the robustness of the procedures, validating the program through confirmation of its results with a different sample and in a different year (hence minimizing the possibility that results were due to aspects such as chance, or particular characteristics of the 2008 group, or other circumstances occurring during that particular year, such as faculty’s initial motivation). They also show the stability of the instruments employed, which adds to their potential for comparative use (in 22 evaluations, only one registered a comparatively substantial difference, i.e., the 2008 score by SPs at T0, when compared with their 2009 score at T0).

The fact that SPs’ ratings at T0 are lower in 2009 (close to faculty’s ratings) than in 2008 may partly be an effect of learning. Throughout their first year, SPs were exposed to professionals increasingly more effective at communicating, which may have created new (and higher) standards for them, then applied to the next year’s participants at T0. They may also have become more familiarized with the specificities of the competences taught, moving away from the global ways in which patients tend to evaluate health professionals [34-36] and providing more focused assessments at T0. These possibilities are in line with research indicating that non-trained raters tend to give students higher scores than trained raters [37,38].

On the other hand, participants in the 2009 class scored lower at T0 than those of the 2008 group in all measures. This could indicate that the 2009 group began with fewer communication skills than did the 2008 sample (to which SPs would have been sensitive, with their ratings). However, the generally smaller standard deviations in 2009 for SPs (as well as for external observers) support the learning hypothesis (Table 2). Still, the standard deviation for SPs at T0 is rather large (and larger than the 2008 one), and further studies on how these patients – as well as RPs – assess their health professionals are needed for a more thorough understanding of these results.

### Comparison between standardized and real patients

Regarding the second goal of this study, the analyses indicate that communication competences acquired and used throughout the program, and applied with SPs, were effectively transferred to situations with RPs. These results confirm previous research indicating that after-program improvements in students’ communication skills are observed both in SPs’ and in RPs’ interviews [10,11,21,22]. The similarity of the results (with statistically non-significant differences) between the two groups reinforces the notion that SPs constitute effective proxies for RPs [15].

One difficulty of using the SEGUE framework in real situations, though, had to do with the duration of the interviews. Within the 25-minute interviews, the parameters of the SEGUE framework applied to encounters with SPs in controlled situations more easily than to encounters with RPs. This observation is in line with the idea that SPs may not provide enough realism in clinical interviews, and experienced clinicians may deviate from checklists while employing adequate interviewing skills [25]. Interviews with some RPs required attention and response to several relevant and emotionally-loaded situations that took most of the interview time, preventing some items of its structure from being discussed within that time frame. Whenever these competencies were correctly used (and indeed necessary in such cases), and given the lack of items in the SEGUE framework to be checked regarding this special attention to the person, “being personally present”, and flexibility [34], our choice was to mark as “non applicable” parts of the interview that were not covered. This adaptation may partly explain the slightly higher scores participants obtained with RPs than with SPs (contrary to previous research [23]). For RPs, the number of items marked “yes” was, in several cases, closer to the total number of valid items (those considered “applicable”).

One limitation in this study is that it did not include interviews with RPs at T0, preventing analyses on the magnitude of the changes with the program for those patients. Also, since students voluntarily enroll in this program, they may be particularly motivated to learn and apply these communication skills, limiting the generalization of program effects to other healthcare professionals. Despite these limitations, the study has the advantages of complementing participants’ (subjective) self-assessments with evaluations by external observers and by SPs; including evaluations at different points in time, namely before and after the program; using two different samples of participants; and including SPs in controlled situations, as well as RPs in real situations. Future studies with control groups and participant randomization in a pretest-posttest design can help disentangle the effects of confounding variables, such as participants’ own experience, or the effects of the more advanced classes of the program, on the observed increase in basic communication skills and in self-confidence. Once the outcomes of the overall program are established, its defined components can also be manipulated to assess their specific effects in the results. Qualitative interviews will provide relevant information on participants’ perspectives about what makes the communication training effective and why. Finally, the program’s robustness can further be tested when taught by different instructors in future studies.

## Conclusions

The consistent positive results in this study indicate that this type of program structure and procedures can be used to improve communication skills among healthcare professionals. The skills learned and practiced within the program in artificial settings are transferrable to controlled situations with simulated patients, and results are replicated in different samples of participants. They also transfer to real-life situations with real patients, the ultimate goal of these courses. Future follow-up studies with these samples will assess the permanence over time of these communication skills in clinical interviews.

## Competing interests

The authors declare that they have no competing interests.

## Authors’ contributions

All authors participated in the conception of the study after RMC’s initial proposal, and in the data collection process. IPC, VGP and FRS conducted the literature review and IPC analyzed the data. IPC wrote a first draft of the manuscript, which was read by the other authors. All authors contributed to the paper’s final version. All authors read and approved the final manuscript.

## Author’s information

Parts of this work were presented at the International Conference on Communication in Healthcare in Verona, Italy, September 2010.

## Pre-publication history

The pre-publication history for this paper can be accessed here:

http://www.biomedcentral.com/1472-6920/14/92/prepub
